# Phylogeography and population genetic structure of the European roe deer in Switzerland following recent recolonization

**DOI:** 10.1002/ece3.8626

**Published:** 2022-02-19

**Authors:** Nina Vasiljevic, Nadja V. Morf, Josef Senn, Sílvia Pérez‐Espona, Federica Mattucci, Nadia Mucci, Gaia Moore‐Jones, Simone Roberto Rolando Pisano, Adelgunde Kratzer, Rob Ogden

**Affiliations:** ^1^ Zurich Institute of Forensic Medicine University of Zurich Switzerland; ^2^ Swiss Federal Research Institute WSL Birmensdorf Switzerland; ^3^ Royal (Dick) School of Veterinary Studies and the Roslin Institute University of Edinburgh Midlothian UK; ^4^ ISPRA‐Istituto Superiore per la Protezione e la Ricerca Ambientale Area per la Genetica della Conservazione BIO‐CGE Bologna Italy; ^5^ Institute for Fish and Wildlife Health (FIWI), Department of Infectious Diseases and Pathobiology, Vetsuisse‐Faculty University of Bern Bern Switzerland

**Keywords:** conservation genetics, gene flow, microsatellites, mtDNA, phylogeography, population structure, ungulate management

## Abstract

In the early 1800s, the European roe deer (*Capreolus capreolus*) was probably extirpated from Switzerland, due to overhunting and deforestation. After a federal law was enacted in 1875 to protect lactating females and young, and limiting the hunting season, the roe deer successfully recovered and recolonized Switzerland. In this study, we use mitochondrial DNA and nuclear DNA markers to investigate the recolonization and assess contemporary genetic structure in relation to broad topographic features, in order to understand underlying ecological processes, inform future roe deer management strategies, and explore the opportunity for development of forensic traceability tools. The results concerning the recolonization origin support natural, multidirectional immigration from neighboring countries. We further demonstrate that there is evidence of weak genetic differentiation within Switzerland among topographic regions. Finally, we conclude that the genetic data support the recognition of a single roe deer management unit within Switzerland, within which there is a potential for broad‐scale geographic origin assignment using nuclear markers to support law enforcement.

## Graphical abstract

After 19th century extirpation, the European roe deer naturally recolonized Switzerland. Using mitochondrial and nuclear DNA markers, we demonstrate that Swiss roe deer had natural, multidirectional immigration from neighboring countries and that there is evidence of weak genetic differentiation within Switzerland among topographic regions.

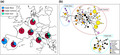

## 1 INTRODUCTION

### 1.1 Genetic variation and natural recolonization of wildlife populations

Biodiversity and genetic variation within species are shaped by both natural and anthropogenic factors. Within Europe, climate change during the last glacial period resulted in the creation of three main refugia on Mediterranean peninsulas from where different taxa later recolonized central and northern Europe through range expansion (Hewitt, 1999). More recently, human actions such as deforestation, habitat fragmentation, infrastructure development, and overexploitation of wildlife populations, for example, through illegal hunting, have affected species distributions, caused population declines, and even led to local or Europe‐wide extinctions (Deinet et al., 2013). Despite these historic and contemporary population declines, there is also evidence of recent population increases and range expansions for many of the larger bird and mammal species (Deinet et al., 2013). However, once reduced and fragmented, populations are likely to be recovering with diminished genetic diversity and often face poor genetic connectivity (Frankham, 1996). This was the case for the Alpine ibex (*Capra ibex ibex*) that underwent stepwise re‐introductions from very small founder populations that had been subjected to serial bottleneck events (Biebach & Keller, 2010) and where high levels of inbreeding are reducing long‐term population growth (Bozzuto et al., 2019). Aside from human interventions, species often naturally restore their population sizes as they disperse and recolonize previously inhabited territories (Sommer & Nadachowski, 2006). One of the many successful natural recolonizations is the comeback of once critically endangered gray wolf (*Canis lupus*) in central and western Europe (Chapron, 2014). Such dynamic range expansion led to the establishment of a new wolf population in Central European lowlands (Poland), characterized by lower microsatellite and mtDNA genetic diversity in comparison to Baltic and Carpathian wolf populations (Szewczyk et al., 2019). In general, the reasons for the natural recolonization success range from coordinated legal protection, controlled hunting, habitat management, and site protection (Sanderson & Harris, 2020). Understanding the demographic and genetic consequences of these anthropogenic and natural processes and their interactions is necessary to predict the future of populations and establish successful conservation and management practices.

### 1.2 Roe deer demographic and life history in Europe

The European roe deer (*Capreolus capreolus*) is one of the most abundant wild ungulates and an important hunting species that is distributed across the European continent from the Mediterranean to Scandinavia (Apollonio et al., 2010). It is a highly adaptable species and its widespread distribution is due to its success in colonizing different environments and habitats (Andersen et al., 1998; Hewison et al., 1998). A complex contemporary pattern of mitochondrial DNA population structuring probably results from Pleistocene isolation in the main southern glacial refuges, resulting in a deep divergence between these lineages and recent extinctions and translocations (Randi et al., 2004). Randi et al. (2004) showed that phylogenetic trees and networks identified three main mtDNA main groups, corresponding to Clade East, West, and Central. These three main haplogroups could have originated in Iberia (Clade West and perhaps Central) or in the Balkans (Clade East). The Clade East haplotypes have a limited distribution, which supports the existence of an eastern glacial refuge, from which roe deer did not disperse far westward. Conversely to the Clade East, the widespread distribution of Clade Central haplotypes in the Balkans, central and northern Europe, Apennines, Alps, and Iberia supports the existence of distinct refugial populations that contributed substantially to the recolonization of Europe.

Thus, roe deer likely recolonized Western Europe after the last glacial maximum from three different refugia: Iberia, Italy, and the Balkans. This pattern is consistent with the reconstructed evolutionary history of several plant and animal species in Europe (Hewitt, 1999; Taberlet et al., 1998). Prior to the 19th century, deforestation, intensification of agriculture, uncontrolled hunting, and other anthropogenic activities have affected the distribution of natural populations of roe deer in Western Europe (Danilkin & Hewison, 1996). In some regions, declines were more severe; for example, the range of roe deer in Britain was reduced to a few relict populations in the Scottish highlands (Ritchie, 1920) and a remnant population of only around 100 roe deer remained in the southernmost area of Sweden (Thulin, 2006).

Regarding the life history, roe deer population social organization varies through the year. Generally, the majority of roe deer are observed alone or in small family groups during summer (pairs usually comprise a doe and her kid, two kids, or a rutting adult buck with a doe). The situation is somewhat different in winter, when larger groups of four or more animals are most commonly observed.

Roe deer do not maintain exclusive territories but live within overlapping summer and winter home ranges. In summer, which is the period of territoriality and reproductive activity, the deer which are primarily solitary are dispersed over the population range.

Adult bucks establish summer territories which they have to re‐establish each year in contest with other bucks. In April and May, prior to giving birth, it does separate from their groups and occupy kidding ranges which remain same from year to year and measure several hectares.

In contrast, during winter when bucks are no longer territorial, the deer mainly form groups which are concentrated on feeding ranges. Females with kids return to their winter home ranges between October and December (Danilkin & Hewison, 1996).

### 1.3 Roe deer demographic history in Switzerland and hunting laws

Even more drastic declines happened in Switzerland, where after the Napoleonic wars, crop failures and resulting famines led to uncontrolled hunting, which combined with subsequent cattle browsing competition, caused roe deer to effectively disappear from the country by around 1800 (Apollonio et al., 2010). Roe deer may have been extinct or numbers extremely low until 1875, when a federal hunting law introduced wildlife conservation and controlled hunting in Switzerland (Müller, 2005). This resulted in rapid recolonization of Switzerland by the roe deer considered to be from Germany and France (Breitenmoser, 1998). In addition, deliberate translocations of roe deer from unknown sources were thought to have been carried out at the end of the 19^th^ century, particularly in the southern part of Switzerland (Apollonio et al., 2010). The roe deer is now the most common ungulate species in Switzerland, with a stable population of more than 130,000 individuals (BAFU, 2017). With the successful recolonization and increased population numbers, a Federal Hunting Law was enacted in 1986, where the objective shifted from the protection of animals to the protection of forests and the control of populations. This law requires the 26 cantons to establish management strategies for all huntable species, which can create difficulties, as there are different ways of managing wildlife on such a small territory (41,285 km^2^), with animals moving freely among them. In order to address the intricacies of current fine‐scale canton‐level management and improve cooperative management plans, it is important to understand roe deer population structure and connectivity across the landscape.

The topography of Switzerland is varied and mainly consists of a high mountainous region (the Alps) in the south and the Jura Mountains in the northwest connected by the Central Plateau with rolling hills and plains. Those three different topographic regions may influence the distribution and movement of Swiss roe deer across the country. In addition, several large lakes are scattered throughout the country which can further curb dispersal and gene flow in roe deer.

### 1.4 Conservation genetic management of roe deer

Conservation genetics typically uses molecular markers to enable measurement of genetic diversity, gene flow, and spatial population genetic structure. The resulting data can, in turn, be used to understand phylogeography, define evolutionary significant conservation units, and identify the origins of unknown samples. Because of its high mutation rate, haploid maternal inheritance, and lack of recombination, the hypervariable mitochondrial DNA (mtDNA) control region is often used as a marker in investigations of species’ population genetic diversity (Avise et al., 1987). It offers increased sensitivity for inferring recently evolved population genetic structure relative to other molecular markers (Zink & Barrowclough, 2008) and is a potentially useful tool to address questions relating to the phylogeography of roe deer in Switzerland. More specifically, comparative genetic analysis of mtDNA control region haplotypes within Switzerland and among surrounding countries should shed light on the historic recolonization of roe deer through analysis of the evolutionary footprints left on their contemporary geographic distribution.

To complement measures of mitochondrial DNA diversity, nuclear genetic markers that show high polymorphism can be used to provide finer‐scale population resolution and reveal familial or individual‐level variation. While single‐nucleotide polymorphism (SNP) markers may be analyzed at scale throughout the genome, the greater allelic diversity at microsatellite loci means that they remain a highly effective tool for examining neutral variation in landscape‐scale population genetic studies (Hodel et al., 2016). The frequency of the microsatellite alleles observed in a population can be used to characterize its neutral genetic structure and to assess the probability of a sample originating from that area. Such probabilistic approaches have important implications for wildlife management and forensic application. Estimation of genetic structure and understanding connectivity among populations help to identify management units (MU) (Moritz, 1994) that are typically used to delineate entities for monitoring and to regulate the effects of human activity on species demography. These data are also used to examine the functionality of wildlife corridors to ensure genetic and demographic connectivity (Rabinowitz & Zeller, 2010), as well as to inform conservation translocations (Moritz, 1999).

A few studies have explored the effect of transportation infrastructure on the genetic structure and diversity of roe deer populations in North‐Central Switzerland (Hepenstrick et al., 2012; Kuehn et al., 2007), indicating that fenced motorways present a barrier to gene flow between local roe deer populations leading to population differentiation, but that fragmentation has not yet affected genetic diversity within those populations. These studies showed that genetic analyses can be important tools to investigate landscape relevant issues, such as the effect of anthropogenic barriers on animal populations and their migration routes, and support the design and the functionality of wildlife corridors to sustain landscape connectivity.

However, what remains largely unexplored is the recolonization history, phylogeography, and contemporary population genetic structure of naturally returning roe deer populations across Switzerland as a whole. In particular, the Central Swiss Alps, representing a potentially strong geographic barrier to gene flow, might be expected to form a pronounced north‐south divide, with implications for future management plans and regulations in Switzerland.

Alongside wildlife management, a greater understanding of population genetic and geographic structuring provides the principal basis for individual geographic assignment, ultimately supporting the development of individual traceability tools. Assigning individuals to a particular source population has proved to be very useful as a conservation management tool, for example, in assigning individual salmon to spawning river in Alaska (Smith et al., 2005) or assigning Sumatran orangutan to their population of origin on the island (Rianti et al., 2015). While such information would also have relevance to roe deer management, assignment testing is likely to prove more useful in tackling illegal hunting. Wildlife forensic applications of geographic assignment, to determine whether an animal trophy originates from a protected population rather than the legal source, are gaining popularity (Ogden & Linacre, 2015) and create a clear opportunity to support the enforcement of roe deer hunting regulations in Switzerland.

### 1.5 Study aims

In this study, we use a classical conservation genetic approach to investigate the natural recolonization of the European roe deer (*Capreolus capreolus*) in Switzerland and assess contemporary population structure in relation to landscape features, in order to understand underlying ecological processes and inform future roe deer management strategies.

More specifically we ask: (i) Where did roe deer recolonize Switzerland from?; (ii) what patterns of population contemporary genetic structure and gene flow can be observed in Swiss roe deer?; and (iii) are there any clear implications for conservation management and opportunities for development of forensic traceability tools?

## 2 MATERIALS AND METHODS

### 2.1 Sampling and DNA extraction

A total of 319 roe deer samples were obtained from Switzerland and Italy. Roe deer samples (*n* = 279; blood = 226 and tissue = 53) were collected in Switzerland between 2004 and 2017, by local hunters. Additional tissue samples were collected from Northern Italy close to the Swiss border (Lombardy; *n* = 28) and Central Italy (Emilia‐Romagna region; *n* = 12). Samples were grouped into six regions based on broad landscape features, primarily according to topographic variation (Figure S1), as follows: Swiss regions: North, *n* = 25; Central‐East, *n* = 106; Central‐West, *n* = 102; South‐West, *n* = 20; South‐East, *n* = 54 (26 Swiss and 28 Northern Italian samples). The additional 28 samples from Lombardy were included in the southeastern Swiss region as they originated close to the Swiss border with no barrier to free movement. We decided to split the Central Plateau into two localities, an eastern and western one because of the great distance between two ends of the Plateau and because of presence of several big lakes and highways that transect the Plateau and pose an obstacle to roe deer free movement. Lastly, we wanted to enable detection of immigration from different sides of Switzerland. The sixth group consisted of 12 individuals from central Italy, resulting in a total of 319 samples. DNA from tissue and blood samples was extracted using the DNeasy^®^ Blood and Tissue kit (Qiagen). DNA from blood swab samples was extracted using a chelex protocol followed by a Qiagen PCR purification kit (Qiagen, Switzerland) as described in Morf et al. (2021).

### 2.2 Genotyping and mitochondrial DNA sequencing

A representative subset (*n* = 152) of the available samples were randomly selected for mtDNA sequencing in order to ensure that each of the five regions in Switzerland and one in Central Italy was sufficiently represented (North, *n* = 18; Central‐East, *n* = 44; Central‐West, *n* = 24; South‐West, *n* = 17; South‐East, *n* = 37 and Central Italy, *n* = 12). A single 704‐bp long mitochondrial DNA (mtDNA) control region fragment was amplified using two primers developed by Randi et al. (1998): L‐Pro 5’CGT CAG TCT CAC CAT CAA CCC CCA AAC C‐3’ and H‐Phe 5’‐ GGG AGA CTC ATC TAG GCA TTT TCA GTG‐3’. PCRs (20 µl total volume) contained 0.5 µl (10 µM) each primer, 12.5 µl DreamTaq PCR Master Mix (2×) (Thermo Fisher Scientific) or HotStartTaq Master Mix (Qiagen) with cycle conditions: 95°C for 5 min; 35 cycles at 94°C for 30 s, 62°C for 30 s, 72°C for 1 min; 72°C for 10 min. PCR products were purified using ExoSAP‐IT™ (Thermo Fisher Scientific). Samples were subjected to bidirectional Sanger sequencing with BigDye™ Terminator v3.1 Cycle Sequencing Kit (Thermo Fisher Scientific) using the L‐Pro and H‐Phe PCR primers and two internal primers Lcap362 and Hcap493 (Randi et al., 1998), which overlap to generate four sequence reads for each sample. Sequences were resolved on an ABI 3730 genetic analyzer (Thermo Fisher Scientific). Sequences were edited by eye and aligned with published European roe deer control region sequences from Portugal, Spain, France, Germany, Italy, and Serbia (GenBank accession numbers AY625752 ‐ AY625876, *n* = 76) from Randi et al. (2004) using Geneious 11.1.5 (BioMatters Inc, New Zealand).

A total of 316 samples were genotyped using a species‐specific microsatellite panel consisting of 13 tetrameric microsatellites and two sex‐specific markers shown to be polymorphic, following the method described in (Morf et al., 2021). The PCR products were resolved using an ABI 3100 Genetic Analyzer (Thermo Fisher Scientific) and allele sizes called using GeneMapper ID‐X version 1.4.

### 2.3 Data analysis

#### 2.3.1 Mitochondrial DNA data

To evaluate lineage diversity in roe deer populations in Switzerland and compare to the data available from previous study Randi et al. (2004), unique haplotypes were identified using DnaSP, version 6.12.01 (Rozas et al., 2017). Newly observed haplotypes were named following the numbering system of Randi et al., 2004. Rarefied haplotype diversity (Hr), nucleotide diversity (average over loci, π), and average pairwise nucleotide substitutions (*k*) between haplotypes in each region were calculated in ARLEQUIN, version 3.5 (Excoffier & Lischer, 2010).

Distributions of mtDNA haplotypes were examined across neighboring countries and within Switzerland by plotting haplotypes onto location maps of Europe and Switzerland. The relationship among haplotypes was examined by constructing a median‐joining network implemented in the program PopART (Bandelt et al., 1999; Leigh & Bryant, 2015).

### 2.4 Microsatellite data

#### 2.4.1 Genetic diversity, Hardy–Weinberg equilibrium, and linkage disequilibrium

The software MicroChecker, version 2.2.3 (Van Oosterhout et al., 2004), was used to test microsatellite loci for null alleles, allele dropout, and scoring errors due to stutter peaks. Standard indices of genetic diversity, including number of alleles (*N*_A_), observed (*H*_o_), and expected (*H*_e_) heterozygosity across all microsatellite loci and regions, were computed using the package adegenet, version 2.1.1 (Jombart, 2008), in R, version 3.5.3 (R Core Team, 2014). We also calculated rarefied allelic richness (*A*_R_) and inbreeding coefficients with FSTAT, version 2.9.4 (Goudet, 2003). Allelic richness was calculated as it yields a measure of allelic diversity corrected for differences in samples size. Conformation to Hardy–Weinberg equilibrium (HWE) and linkage disequilibrium (LD) were tested by performing Fisher's exact tests in GENEPOP, version 4.2 (Rousset, 2008), with default settings for Markov chain parameters.

#### 2.4.2 Population genetic structure

The program STRUCTURE, version 2.3.4 (Pritchard et al., 2000), was used to infer the putative number of populations (*K*) based on microsatellite data. The Bayesian clustering method in STRUCTURE attempts to identify genetically distinct subpopulations on the basis of patterns of allele frequencies. A series of ten independent runs per *K* (ranging 1 to 10) were performed using 1,000,000 MCMC iterations after a burn‐in of 500,000 replicates, using an admixture model with correlated allele frequencies without prior sampling information.

Based on the initial results from STRUCTURE that indicated very weak population structure, another set of analyses was performed using prior information on sampling location (LOCPRIOR) as suggested for datasets with few markers, few individuals, or very weak structure (Pritchard et al., 2010). A series of six independent runs per *K* value (*K* = 1–6) were performed at 500,000 MCMC iterations after a burn‐in of 200,000 replicates. The burn‐in and data collection iterations, and the number of replicates, were reduced in this phase, based on observed consistency among replicates, in order to reduce computation time. Results were summarized using STRUCTURE HARVESTER (Earl, 2012). Both log‐likelihood plots and the delta *K* (Evanno et al., 2005) statistic were used, with caution, to infer the optimal *K* value, given the known difficulties that may arise in using these methods (Gilbert, 2016).

To complement the STRUCTURE analysis and to assess the degree to which these five Swiss selected sampling regions differ from each other when adopting an approach without assumptions about HWE or LD, we conducted a discriminant analysis of principal components (DAPC) (Jombart et al., 2010) in *adegenet* package (Jombart, 2008) for the R, version 3.5.3. (R Core Team, 2014). This method aims to identify and describe the relationship between genetic clusters. A multivariate DAPC analysis performs a preliminary data transformation step using principal component analysis (PCA) to create uncorrelated variables that summarize total variability (within and between groups). These variables are then used as input to DA, which aims to maximize between‐group variability while minimizing variation within groups and achieve the best discrimination of genotypes into predefined clusters. For this DAPC, forty principal components of PCA and all (4) discriminant functions were retained. The function *find*.*clusters* was used to identify the number of clusters (*K*) in our data and to compare with the prior groups (regions). The optimal clustering solution corresponds to the lowest Bayesian information criterion (BIC), and in this case, it clearly indicated 5 clusters (*K* = 5) that we decided to retain.

To determine the level of genetic differentiation between pairs of populations, F‐statistics (Weir & Cockerham, 1984) were calculated using ARLEQUIN, version 3.5.2 (Excoffier & Lischer, 2010). *F*_st_ values are obtained from variance in microsatellite allele frequencies. To inspect the population differentiation, we also performed an analysis of molecular variance (AMOVA) in ARLEQUIN with five regional populations nested within northern (North, Central East, Central West) and southern (South East and South West) Swiss groupings. Finally, isolation by distance (IBD) patterns based on microsatellites genotypes were examined to determine association between matrices of pairwise codominant genotypic and geographic (Euclidean) distances by performing a simple Mantel test (1000 permutations) visualized in adegenet, version 2.1.1 (Jombart, 2008), in R, version 3.5.3 (R Core Team, 2014).

## 3 RESULTS

### 3.1 Mitochondrial DNA markers

The mtDNA alignment using 228 sequences in total (152 from our study and 76 haplotype sequences from GenBank) and 704 nucleotides showed a total of 89 haplotypes including twelve novel sequences (H161‐H173) in Switzerland (GenBank accession nos MW916295‐MW916306) and 47 polymorphic sites.

Each haplotype was observed from 1 to 69 times (distributions are represented in Table S1). Haplotype diversity across all European countries in this study was high (*H* = 0.97 ± 0.002), but nucleotide diversity (π = 0.01 ± 0.006) and pairwise divergence (*k* = 7.54 ± 3.52) were low (Table 1).

**TABLE 1:** Estimates of mtDNA control region diversity statistics for roe deer samples by country/region

	*N*	Nh	Hr (*SD*)	π (*SD*)	*k*
Portugal	23	6	0.81 (0.041)	0.008 (0.004)	5.36
Spain	53	12	0.86 (0.033)	0.009 (0.05)	6.79
France	12	5	0.85 (0.067)	0.005 (0.003)	3.52
Germany	15	8	0.84 (0.085)	0.005 (0.003)	4.11
Switzerland	140	26	0.93 (0.007)	0.009 (0.005)	6.25
Switzerland North	18	13	0.96 (0.03)	0.008 (0.005)	5.89
Switzerland Central‐East	44	10	0.79 (0.041)	0.007 (0.004)	5.30
Switzerland Central‐West	24	10	0.87 (0.045)	0.007 (0.004)	4.79
Switzerland Southwest	17	7	0.89 (0.036)	0.011 (0.006)	7.47
Switzerland Southeast	37	14	0.89 (0.027)	0.009 (0.005)	6.85
Italy	351	27	0.89 (0.008)	0.009 (0.005)	6.52
Serbia	178	38	0.96 (0.004)	0.01 (0.005)	7.33
Average/Total	772	89	0.97 (0.002)	0.01 (0.006)	7.54

Abbreviations: Hr, rarefied haplotypic diversity (*SD*); *k*, average number of pairwise differences; *N*, number of mtDNA sequences; Nh, number of haplotypes; π, nucleotide diversity (*SD*).

### 3.2 European level analysis of roe deer haplotype diversity with respect to Swiss populations

The mean haplotype diversity across all regions in Switzerland was high (0.93) and comparable to the haplotype diversity of Serbia (0.96) which has the highest haplotype diversity among European countries observed to date. The lowest diversity among European countries was found in Portugal (0.81). On a smaller scale, haplotype diversity within Switzerland (Table 1) was greatest in the Northern region (0.96) and lowest in the Central‐East region (0.79).

**FIGURE 1:**
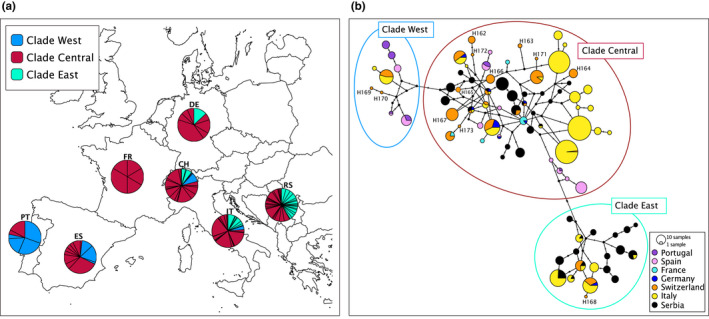
(a) The proportion of mtDNA clades in European countries of sampled roe deer populations. Pie charts indicate the proportion of mtDNA control region haplotypes belonging to the Central, Western, and Eastern Clades in countries: PT‐Portugal; ES‐Spain; FR‐France; DE‐Germany; CH‐Switzerland; IT‐Italy; RS‐Serbia. In the European context, all three clades are represented in Switzerland, which is dominated by the Central Clade. Represented haplotypes are based on 704 base pairs of the mtDNA control region and clades are presented in different colors (see legend). (b) Median‐joining network of all 89 known haplotypes showing the distribution of Swiss samples (in orange) relative to six other European countries (Portugal, Spain, France, Germany, Italy, and Serbia). Novel haplotypes (H162‐173 from this study) are indicated by numbers, circles are proportional to frequencies and colors correspond to different countries. The map is adapted from Eurostat, EuroGeographics for administrative boundaries

The geographic distribution of haplotypes belonging to three previously described haplogroups, or clades (see Randi et al., 2004), across Europe is shown in Figure 1a. Haplotypes belonging to all three clades were observed in Switzerland, with 79% of individuals assigned to Clade Central.

The resulting median‐joining haplotype network was highly reticulated (Figure 1b), but supported the distinction of the three main groups that correspond to Randi's Clade East, West, and Central (Randi et al., 2004) (Figure 1b). Haplogroup Clade East was mainly composed of haplotypes sampled in Eastern Europe, Serbia, and few haplotypes sampled in Germany, Switzerland, and in Italy. Haplogroup Clade West included the majority of sequences that were collected in Portugal and Spain and included a few sequences from Switzerland and Italy. Finally, haplotypes in Clade Central were widespread in central and northern Europe and Italy, as well as in Portugal, Spain, Switzerland, and Serbia. A proportion of Swiss sequences that were assigned to novel Swiss haplotypes were mainly distributed in the Clade Central (Figure 1b.).

### 3.3 Geographic distribution mtDNA haplotypes within Switzerland

**FIGURE 2:**
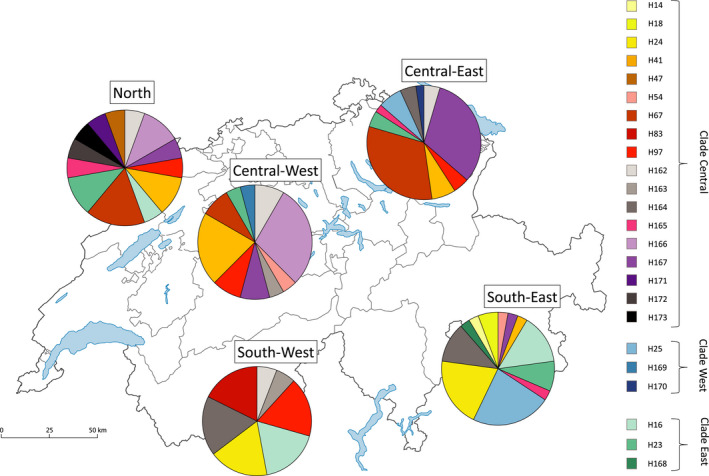
The geographical distribution of roe deer mtDNA control region haplotypes observed in Switzerland. Pie diagrams indicate very different haplotype compositions of populations sampled across Switzerland. The proportion of haplotypes can be differentiated between North and Central and South groups with connectivity through the East and West. All haplotypes are colored and grouped into three clades: Central, West and East (see legend). The map is adapted from Bundesamt fur Statistik (BFS)

On a finer scale, within Switzerland, haplotype distribution did not show a very strong phylogeographic pattern. While the distribution of haplotype variation differs among regions and there are indications of a general pattern that shows distinction north and south of the Alps, there is no clear mitochondrial DNA structuring associated with geography in Switzerland (Figure 2). For example, haplotypes H14, H18, H24, H83, and H168 are present only in southern regions, while H47, H67, H165, H166, and H170 are only in the northern and central regions, but other haplotypes show connectivity between the northern and south eastern regions, for example, H23, H41, and H167. North‐south connectivity through the western side of Switzerland is more evident in the case of haplotypes H16, H97, and H162.

### 3.4 Nuclear microsatellite diversity and population genetic structure

#### 3.4.1 Genetic diversity, Hardy–Weinberg equilibrium, and linkage disequilibrium

A total of 316 samples were successfully genotyped at 13 microsatellite loci, with no evidence of genotyping error observed. Departure from Hardy–Weinberg equilibrium was significant at only one locus (capcap25) in the Central‐West region, following sequential Bonferroni correction (Rice, 1989). No significant linkage was observed, suggesting that all loci are genetically independent. Among the five geographic regions, Swiss roe deer exhibited little variation in genetic diversity, allelic richness was relatively constant ranging from 4.92 to 5.88 alleles per locus and expected heterozygosity ranged from 0.65 to 0.71 between the least and most diverse region (South‐West and North, respectively; Table 2). Values for the inbreeding coefficient, *F*_IS_, were generally low and positive, with the highest value (SE, *F*_IS_ = 0.074) indicating only low levels of localized inbreeding (Table 2).

**TABLE 2:** Measure of genetic variability in Swiss populations of roe deer, as estimated by 13 microsatellite loci

Region	N	*N*_A_	*A*_R_	*F*_IS_	*H*_o_	*H*_e_
North	25	6.1	5.88	−0.008	0.69	0.67
Central‐East	106	6.9	5.7	0.025	0.69	0.71
Central‐West	102	6.5	5.47	0.05	0.63	0.66
South‐West	20	4.9	4.92	0.004	0.67	0.65
South‐East	54	6.1	5.1	0.074	0.63	0.67
Average (*SD*)	307^a^	6.1 (0.75)	5.41 (0.40)	0.029 (0.03)	0.66 (0.03)	0.67 (0.02)

HWE testing was conducted per locus per population; no significant deviations from HWE were observed after sequential Bonferroni correction.

Abbreviations: *A*_R_, rarefied allelic richness; *F*_IS_, inbreeding coefficient; *H*_e_, expected heterozygosity; *H*_o_, observed heterozygosity; *N*, number of genotypes individuals; *N*_A_, average number of alleles.

^a^ Total number of genotyped individuals.

#### 3.4.2 Population genetic structure

**FIGURE 3:**
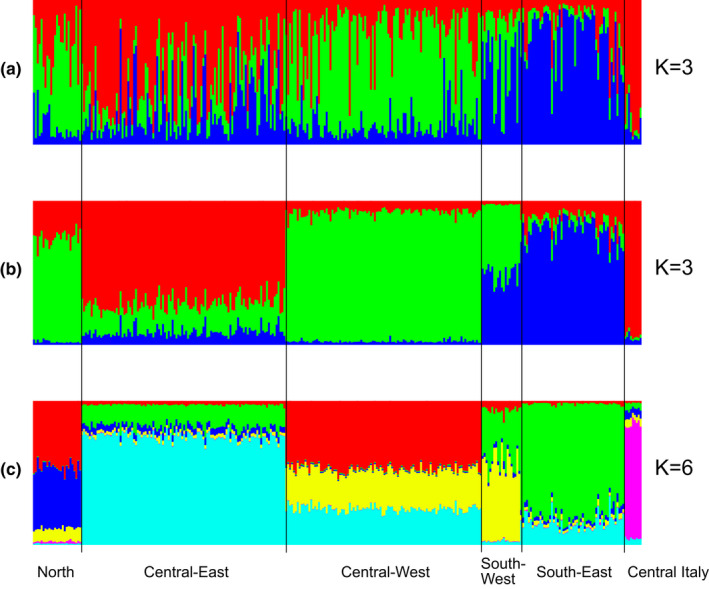
Assignment probabilities of individuals to putative population clusters at (a) *K* = 3 (b) *K* = 3 with prior information on sampling location (LOCPRIOR) (c) *K* = 6 with LOCPRIOR, using the program STRUCTURE 2.3.2. Locations where individuals were sampled are indicated below *x*‐axis

Initial nonspatial Bayesian clustering analysis in STRUCTURE without prior sampling location information for all Swiss roe deer and the central‐Italian outgroup showed weak clustering into three genetic groups (*K* = 3) with varying levels of genetic admixture (Figure 3a) based on posterior probabilities (mean L(*K*)) (Figure S3a). Employing location information as a prior substantially improved clustering at *K* = 3 (Figure 3b and Figure S3b), with one cluster including all individuals from the North and Central West and a second cluster comprised of individuals from the South East. South West individuals were all admixed between these two clusters; the third cluster comprised deer from Central Italy. Individuals from Central East Switzerland were distinct and self‐similar but not strongly assigned to a single cluster. To explore whether there is evidence for population differentiation among the five previously designated topographical regions in Switzerland, representing potentially different management areas, we increased *K* sequentially from *K* = 4 to *K* = 7. At *K* = 6, using locality information for: (i) North (Jura Mountains); (ii) Central West (western Swiss Plateau and west pre‐Alps); (iii) Central East (eastern Swiss Plateau and east Swiss pre‐Alps); (iv) South West (West Swiss Alps); (v) South East (East Swiss Alps and northern Italian Alps); and (vi) Central Italy (Bologna, Modena, Forli Cesena), there was good evidence of geographic structure, as well as introgression associated with geographic neighbors; between the Central East and Central West and to a smaller extent the South East region; and between the North and Central West regions. The Central Italian population was quite distinct with only a very small percentage of admixture with other Swiss populations (Figure 3c).

**FIGURE 4:**
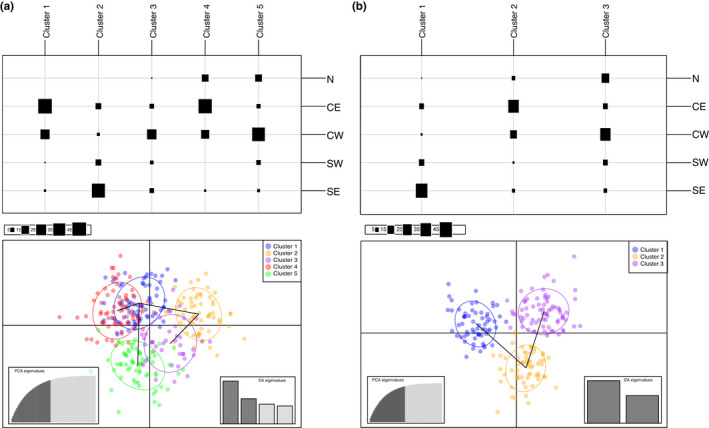
Discriminant analysis of principal components (DAPC) for (a) 307 individuals from Switzerland differentiated into five clusters with a moderate correspondence to five geographic groups (b) reduced dataset of 195 individuals with more equal sample size across groups which shows considerable regional differentiation but only among three clusters. The axes represent the first two linear discriminants (LD). Each dot represents an individual. Numbers represent the different populations identified by DAPC analysis. Geographic groups: N‐North; CE‐Central‐East; CW‐Central‐West; SW‐South‐West; SE‐South‐East

DAPC analysis was conducted to identify genetic clusters within Switzerland and was performed without any *a priori* group assignment and without samples from the distinct Central Italian region. The *find*.*clusters* function retained 100 axes representing more than 88% of the total variance and covered a range of possible genetic clusters from 1 to 30. The lowest BIC value corresponded to K=5. For DAPC analysis, 40 PCA axes and 4 discriminant functions were retained. A high degree of overlap between clusters 1 and 4, and 3 and 5 was evident and showed a similar pattern to that observed in the STRUCTURE results, where North and Central West, then Central East and Central West and finally South West and South East individuals clustered together (Figure 4a).

In a second round of DAPC analysis, we lowered the total sample size in order to equalize the sample sizes among regions. To obtain the optimal number of clusters with the *find*.*clusters* function, we retained 70 axes that represented more than 88% of the total variance. Based on the results the lowest BIC value corresponded to *K* = 4. However, as cluster one and cluster three had a great deal of overlap and formed a single cluster in the scatterplot (see Figure S3), we decided to select the next lowest BIC value (*K* = 3). For DAPC analysis, we retained 40 PCA axes and 2 discriminant functions. Results showed three distinct clusters that more clearly differentiated between southern (cluster one), Central East (cluster two), and Central‐West (cluster three) regions (Figure 4b).

Population‐based gene flow estimates using pairwise *F*_st_ values between 5 Swiss (reduced sample number of 195 individuals) and 1 Central Italian sampling regions indicated significant low to moderate genetic differentiation, with F_ST_ values ranging from 0.02 to 0.14 (Table 3). The highest estimates were observed between roe deer in Central Italy and North West and South West Switzerland. Within Switzerland, the highest estimates were between the South East and North of Switzerland (*F*_ST_ = 0.08) with other pairwise values broadly reflecting relative geographic proximity. The results of isolation by distance testing indicated a significant correlation between genetic and geographic distances when including Central Italy (*r* = .85, *p *= .01), but among Swiss‐only regions this relationship was weaker (*r* = .48) and nonsignificant (*p *= .055).

**TABLE 3:** Pairwise values of *F*_ST_ values (all significant) based on 13 microsatellite loci

Region	(1) N	(2) CE	(3) CW	(4) SW	(5) SE	(6) IT
(1) N						
(2) CE	0.03					
(3) CW	0.02	0.03				
(4) SW	0.05	0.04	0.04			
(5) SE	0.08	0.05	0.07	0.03		
(6) IT	0.11	0.09	0.14	0.13	0.11	

Note

The putative populations used in this analysis were based on the genetic structure analyses (*K* = 5). (1) North, N; (2) Central‐East, CE; (3) Central‐West, CW; (4) South‐West, SW; (5) South‐East, SE; (6) Central Italy, IT.

The AMOVA results testing the hypothesis for hierarchical genetic subdivision within Switzerland, first between northern (non‐Alpine) and south (Alpine) areas of Switzerland and secondly among regions within those areas indicated that most of the variance (91.2%), were attributable to the genetic variation within individuals within sampling sites, whereas only 2.8% of the variance occurred among regions, 2.5% within regions, and 3.6% within populations (all values significant, *p*‐value = .0001). These results show that there is a level of regional genetic differentiation north and south of the Alps which is slightly greater than the level of differentiation among populations within those regions.

## 4 DISCUSSION

Here, we discuss the results of our genetic analyses in the context of two main questions that we posed: What is the recolonization history of roe deer in Switzerland? And what is the contemporary population structure of roe deer in Switzerland? We then examine the implications of these results on current management practices and forensic applications.

### 4.1 Recolonization history

Climate change during the last glacial period resulted in the creation of three main refuge areas in the south of Europe: the Iberian, the Italian, and the Balkans, from where different taxa later recolonized central and northern Europe through range expansion (Hewitt, 1999). This process often resulted in phylogeographic signals within the mitochondrial DNA of many species recolonizing central and northern Europe which can still be observed today.

Within roe deer, the observation of high haplotype diversity but low nucleotide diversity suggests that extant control region mtDNA lineages originated recently despite roe deer populations having a historically large effective size (*N*_e_) (Avise, 2000; Grant & Bowen, 1998). The three mitochondrial DNA clades are observed broadly corresponding to western, central, and eastern Europe (Lorenzini & Lovari, 2006; Randi et al., 2004), and these differences can be used to infer the origin of roe deer recolonizing Switzerland in the past 130 years. Our median‐joining haplotype network analysis depicted the phylogenetic relations among haplotypes sampled in this study and haplotypes previously observed in southern and central European countries. Results showed that the majority of individuals and mtDNA haplotype diversity in Switzerland belonged to the Central Clade, which suggests that historic gene flow occurred across central Europe, particularly in Central Clade, as most haplotypes are connected across countries, while a smaller proportion of individuals with fewer haplotypes belonged to Clades West and East. The presence of haplotypes from all three clades suggests that contemporary recolonization of Switzerland is likely to have involved roe deer immigrating from multiple directions. Although Italy also displays all three clades, our results show that relatively few Central Clade haplotypes were shared between Switzerland and Italy, suggesting that it is unlikely to have been the sole source of Swiss roe deer recolonization. Indeed, historic declines in Italian roe deer also occurred presumably limiting both the number and diversity of individuals available to repopulate Switzerland (Apollonio et al., 2010). Overall levels of mtDNA diversity were high, with 26 of the 89 haplotypes observed in Europe occurring in Switzerland. This further supports the hypothesis that roe deer in Switzerland originated from multiple geographic source populations; however, it is difficult to identify specifically the relative of their contributions. While quantitative comparisons are limited due to varying sampling effort, the fact that 12 haplotypes observed in this study have not been previously observed elsewhere further supports the conclusion that mitochondrial DNA diversity in the recently recolonized Swiss population is at least comparable with other roe deer populations in Europe (Lorenzini & Lovari, 2006; Randi et al., 2004) and shows no signs of single source founder effect. Moreover, these novel haplotypes could represent signals from a remnant Swiss population that was not fully extirpated; however, the available historic demographic data suggest this is unlikely. Instead, we suspect that the novel haplotypes are a consequence of limited sampling effort in neighboring countries during previous studies; the hypervariable nature of the control region requires intense and widespread sampling to confidently conclude that all variation has been observed.

### 4.2 Contemporary population structure within Switzerland

Examination of current population structure within Switzerland based on five Swiss regions: North, Central East, Central West, South West, and South West, indicated no clear mtDNA structuring associated with geography within despite distinct topographical differences between the Jura Mountains, Swiss plateau, and northern and southern Alps. However, analysis of nuclear data revealed three genetic clusters representing the North and Central West, Central East, and the South displaying varying levels of admixture which corresponded to geographic proximity. The fact that only weak clustering was observed when locality information was not included as a prior in the Bayesian analysis emphasizes the lack of strong isolation among regions; this may be due to ongoing gene flow, insufficient divergence time since isolation, or insufficient power in the DNA marker panel, or a combination of these factors. These are the conditions in which prior locality information is recommended for use in STRUCTURE (Pritchard et al., 2000) and the results do nevertheless support the presence of some geographic population structuring within Switzerland. This pattern of genetic differentiation was corroborated by the DAPC analysis, which performed most strongly following equalization of sample sizes, as suggested by Puechmaille (2016) and Wang (2017) for other genetic clustering methods.

The Alps provided the strongest barrier to gene flow according to pairwise F_ST_ results; this result may be readily explained by the ecology of roe deer, for which high mountains and extended periods of snow cover represent strong barriers to movement (Danilkin & Hewison, 1996).

The hypothesis for genetic subdivision between northern and southern Switzerland was further supported by the AMOVA results, which showed a significant proportion of total genetic variance distributed across this north‐south divide. Complementing these findings, the lack of significant association was observed between genetic and geographic distances within Switzerland meaning that the observed genetic variation cannot be explained solely in terms of the geographic distribution of roe deer across a single population.

The presence of weak population structure suggests a dynamic equilibrium between drivers that allow gene flow and those that restrict it. In such a newly established population (100+ years), it is also important to consider the influence of founder history on current population genetic patterns. Here, we discuss the possible causes of the observed weak genetic structure in Swiss roe deer, before considering the management and law enforcement implications.

Our findings indicate that roe deer recolonized from several different directions, raising the possibility that different founder populations would lead to genetic differences in the newly established Swiss population. The idea that roe deer arrived from multiple neighboring countries has being discussed before (Randi et al., 2004; Zachos et al., 2006). Therefore, it would be reasonable to expect that there was some population structure present from the beginning of the recolonization. Following initial recolonization, drivers for maintaining geographic structure distributed in a north‐south direction would include the Swiss Alps, acting as barrier to short‐range migration between northern and southern Switzerland. Similarly, a genetic study on Slovenian roe deer using microsatellite markers showed how natural barriers between mountains and the coastal region represent an important factor limiting gene flow (Buzan et al., 2020). The role of landscape features in affecting the gene flow has been documented in European deer populations, for example, sea lochs, mountain slope elevation, and forest distributions have all been shown to be associated with population genetic structure in Scottish red deer (Pérez‐Espona et al., 2008). Aside from natural environmental barriers to gene flow, anthropogenic barriers in Switzerland such as the dense networks of railways (5300 km) and fenced motorways (1859 km) (www.astra.admin.ch, Swiss Federal Roads Office (FEDRO)) represent other obstacles to free movement of wildlife, with fenced motorways found to cause significant restrictions to gene flow in roe deer (Hepenstrick et al., 2012; Kuehn et al., 2007). Lastly, in terms of life history, roe deer are generally philopatric, with little sex‐biased dispersal (Coulon et al., 2006), and show relatively short dispersal distances of usually less than 5km on maturation (Kurt, 1991; Stubbe, 1990). Taken together, these factors would explain the establishment and maintenance of some genetic structuring within Swiss roe deer.

In contrast, we have shown that despite some population differentiation in Swiss roe deer, regions are not genetically isolated. As a highly adaptable species, they regained their pre‐extirpation distribution quickly and appear to be freely interbreeding with no significant gaps in their distribution allowing for population genetic continuity. While the Swiss Alps are likely to impede gene flow, roe deer may freely disperse along the few valleys that traverse this mountain range. In addition, the creation of wildlife corridors designed to increase functional connectivity across anthropogenic barriers have been shown to be effective in increasing gene flow across the landscape (Burkart et al., 2016). These factors may ultimately disrupt founder population structure and prevent the genetic isolation of roe deer populations.

The transition from traditional nuclear genetic markers to genome‐wide population genetic analyses, such as RADseq approaches or whole‐genome resequencing, raises the possibility of revealing increasingly fine‐scale population structure. This has been demonstrated in conservation genetic management studies of scimitar‐horned oryx (Ogden et al., 2020) and cichlid fishes (Ciezarek et al., 2022), where the introduction of large numbers of SNP markers has enabled population genetic variation to be observed at much greater resolution. However, in the case of Swiss roe deer, while we would expect genome‐wide SNP panels to reinforce our findings of population structure in Switzerland and perhaps detect other regions of differentiation, they would be very unlikely to reverse our interpretation that gene flow persists across these boundaries, negating the creation of multiple genetic management units.

The observed patterns of roe deer genetic variation in Switzerland provide an interesting comparison with the Alpine ibex, the subject of the other main ungulate recolonization in the country. This species was reintroduced from a single Italian founder population, with serial populations then established using small numbers of related founders. Strong genetic founder effects and geographic isolation of new populations, due to the reluctance of this species to cross valleys, means that the Alpine ibex still displays pronounced population genetic structuring and, in many areas, reduced genetic diversity (Biebach & Keller, 2009). In this respect, the genetic results of the Alpine ibex reintroduction and detailed knowledge of its history allow us to infer a very different set of processes and outcomes for roe deer.

### 4.3 Management and forensic implications

Determining how many and where genetically distinct populations exist is fundamental to sound wildlife management (Frankham et al., 2017). Roe deer management regulations in Switzerland are largely based on administrative units without consideration of biological variation.

Here, we aimed to identify and delineate conservation genetic management units that would improve conservation management planning. The weak population structure detected in roe deer suggests some differentiation among the North and Central West, Central East, and the South regions; however, we do not consider the level of genetic variation or geographic separation to be sufficient to define three discrete management units, according to common definitions (Moritz, 1999) and certainly not at the level of 26 administrative units.

In terms of law enforcement, investigations of illegal hunting may involve questions concerning the geographic origin of a roe deer. Based on this study, the level of population structure is insufficient to categorically assign every sample to its region of origin using microsatellite DNA profiling. However, origin assignment in wildlife DNA forensics will typically look to evaluate the relative likelihood of different geographic origins as proposed by the prosecution and defense (Ogden & Linacre, 2015). Under this scenario, it is likely that when comparing the three main genetic regions observed in Switzerland, individual roe deer DNA profiles could be used as evidence to support one proposition over the other.

## 5 CONCLUSION

After being extirpated in the 19th century, roe deer naturally recolonized Switzerland with great success in the 20th century. This study has used molecular genetic markers to investigate this process and its outcomes at a population level, with the overall aim of contributing to a more holistic approach to management planning of this important hunting species.

In answer to our study's first objective, concerning the recolonization origin of Swiss roe deer, we conclude that the evidence supports natural, multidirectional recolonization from neighboring countries.

For the second objective, concerning patterns of contemporary population genetic structure, we conclude that there is evidence of weak genetic differentiation between the North and Central West, Central East and the South regions of Switzerland.

Finally, addressing our third objective, we conclude that the genetic data support the recognition of a single roe deer management unit within Switzerland, within which there is potential for broad‐scale analysis of geographic origin to support law enforcement.

## CONFLICT OF INTEREST

The authors declare no conflict of interest.

## AUTHOR CONTRIBUTION

**Nina Vasiljevic:** Conceptualization (equal); Data curation (lead); Formal analysis (lead); Funding acquisition (supporting); Investigation (lead); Methodology (equal); Software (lead); Visualization (lead); Writing – original draft (equal); Writing – review & editing (equal). **Nadja V. Morf:** Supervision (supporting); Writing – review & editing (supporting). **Josef Senn:** Resources (supporting); Writing – review & editing (supporting). **Sílvia Pérez‐Espona:** Writing – review & editing (supporting). **Federica Mattucci:** Resources (supporting); Writing – review & editing (supporting). **Nadia Mucci:** Resources (supporting); Writing – review & editing (supporting). **Gaia Moore‐Jones:** Resources (supporting); Writing – review & editing (supporting). **Simone Roberto Rolando Pisano:** Resources (supporting); Writing – review & editing (supporting). **Adelgunde Kratzer:** Funding acquisition (lead); Project administration (lead); Resources (lead); Writing – review & editing (supporting). **Rob Ogden:** Conceptualization (equal); Project administration (equal); Resources (supporting); Supervision (lead); Validation (lead); Writing – original draft (equal); Writing – review & editing (equal).

## Supporting information

Appendix S1

## Data Availability

Novel mitochondrial DNA haplotypes have been submitted to GenBank Acc. No.MW916295‐MW916306. The nuclear DNA microsatellite data of all individuals were deposited in Dryad (https://doi.org/10.5061/dryad.fj6q573wq).
