# Supplementary material for: Phylogeography and population genetic structure of the European roe deer in Switzerland following recent recolonization
Source: Ecol Evol. 2022 Feb 19;12(2):e8626. doi: 10.1002/ece3.8626 (PMC8858214; doi:10.1002/ece3.8626)
Supplement: Supplementary file 1 — Appendix S1 [file ECE3-12-e8626-s001.docx]

**Appendix**


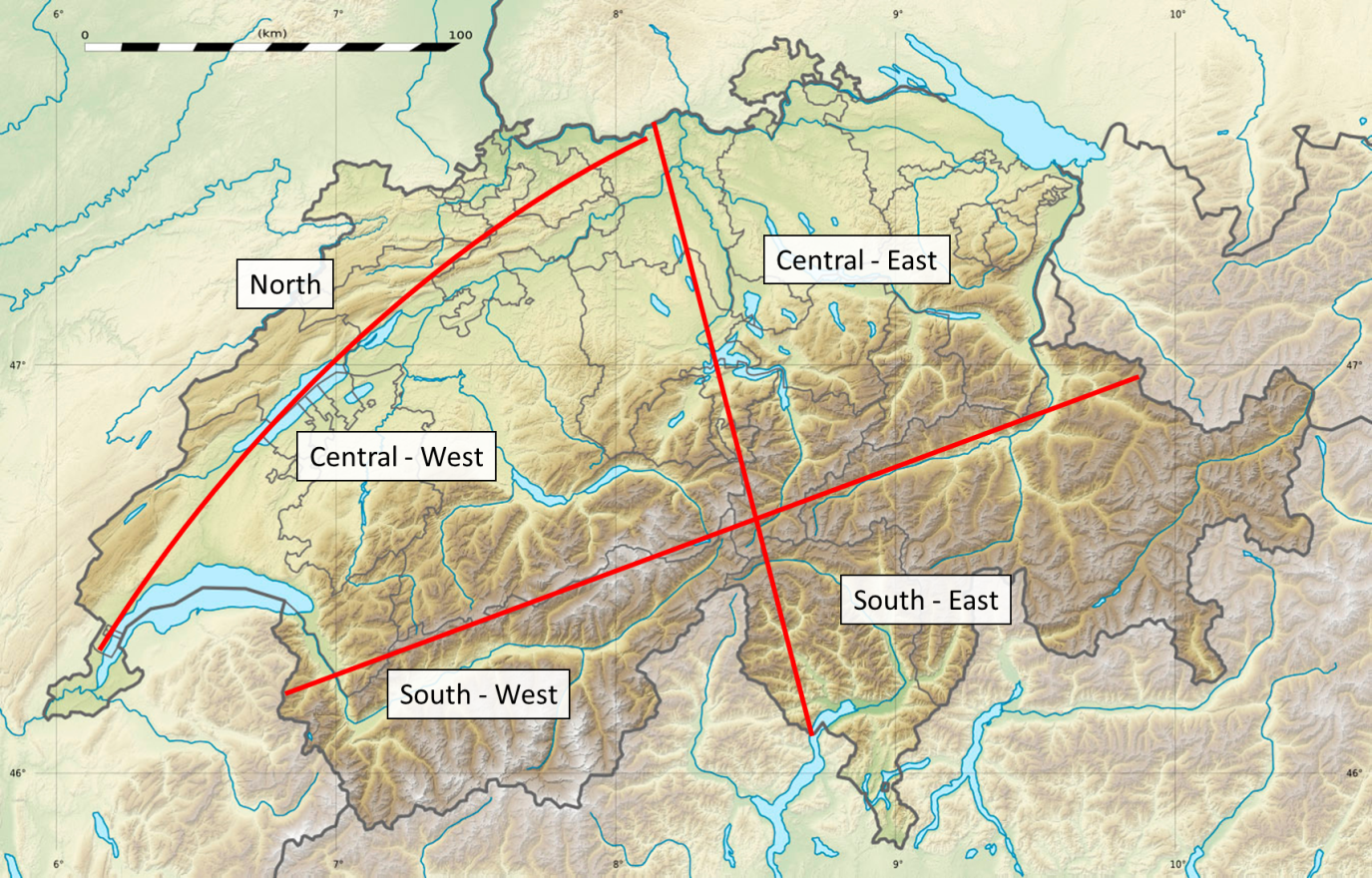


Figure S1. Map of Switzerland with 5 sampling locations based on broad geographic regions relating to underlying topography. The sixth location is in central Italy (Emilia-Romagna region) and not seen on the map. (The map is adapted from: Eric Gaba (Sting - fr:Sting) and NordNordWest, under the [Creative Commons](https://en.wikipedia.org/wiki/en:Creative_Commons) [Attribution-Share Alike 3.0 Unported](https://creativecommons.org/licenses/by-sa/3.0/deed.en) license)


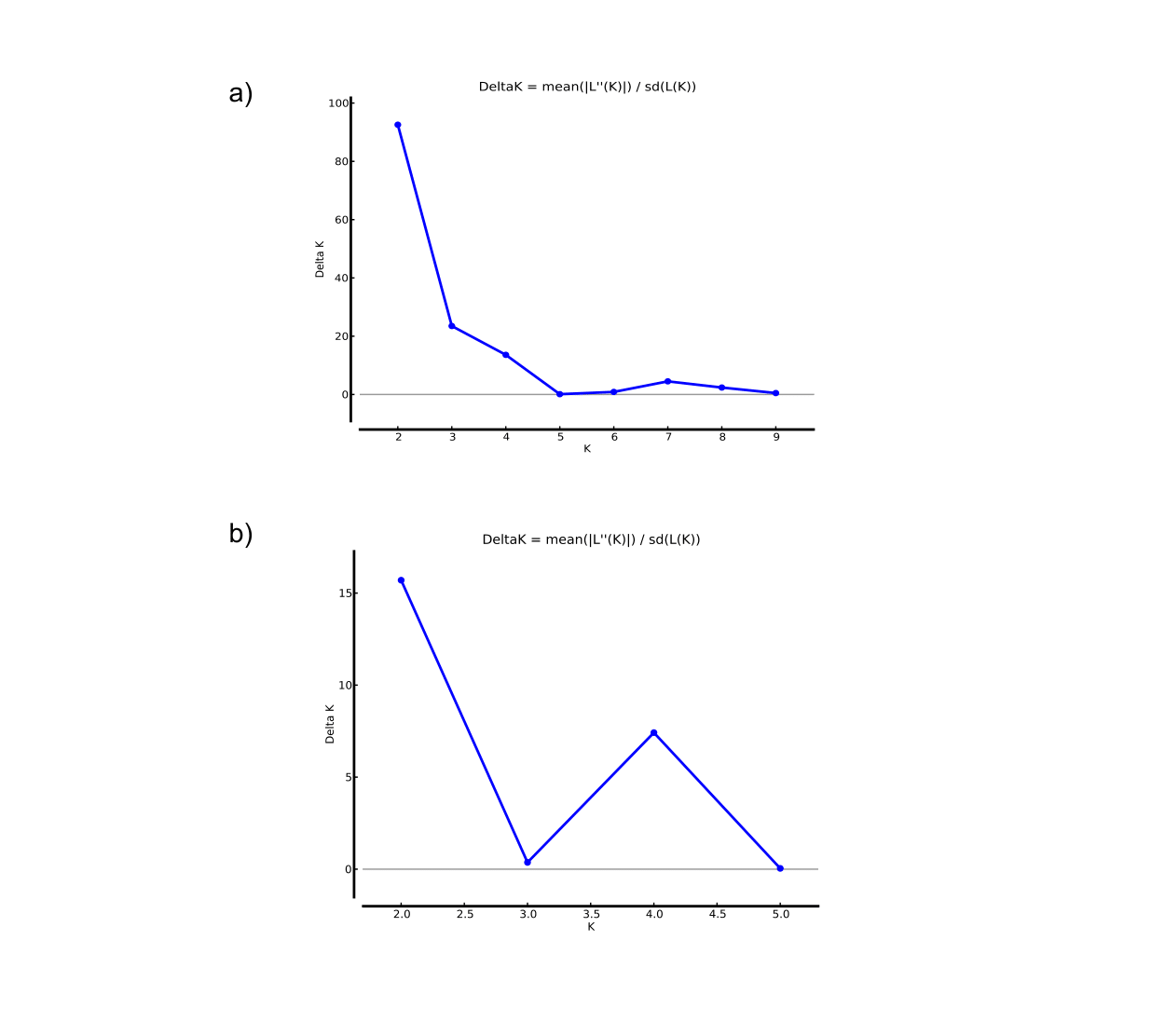


Figure S2. Results for STRUCTURE analysis; values of the statistic "Delta K" for: a) all calculated K-values from 1–10 based on the dataset of nuclear microsatellites without prior information on sampling location (LOCPRIOR), b) for K values 1-6 with LOCPRIOR.


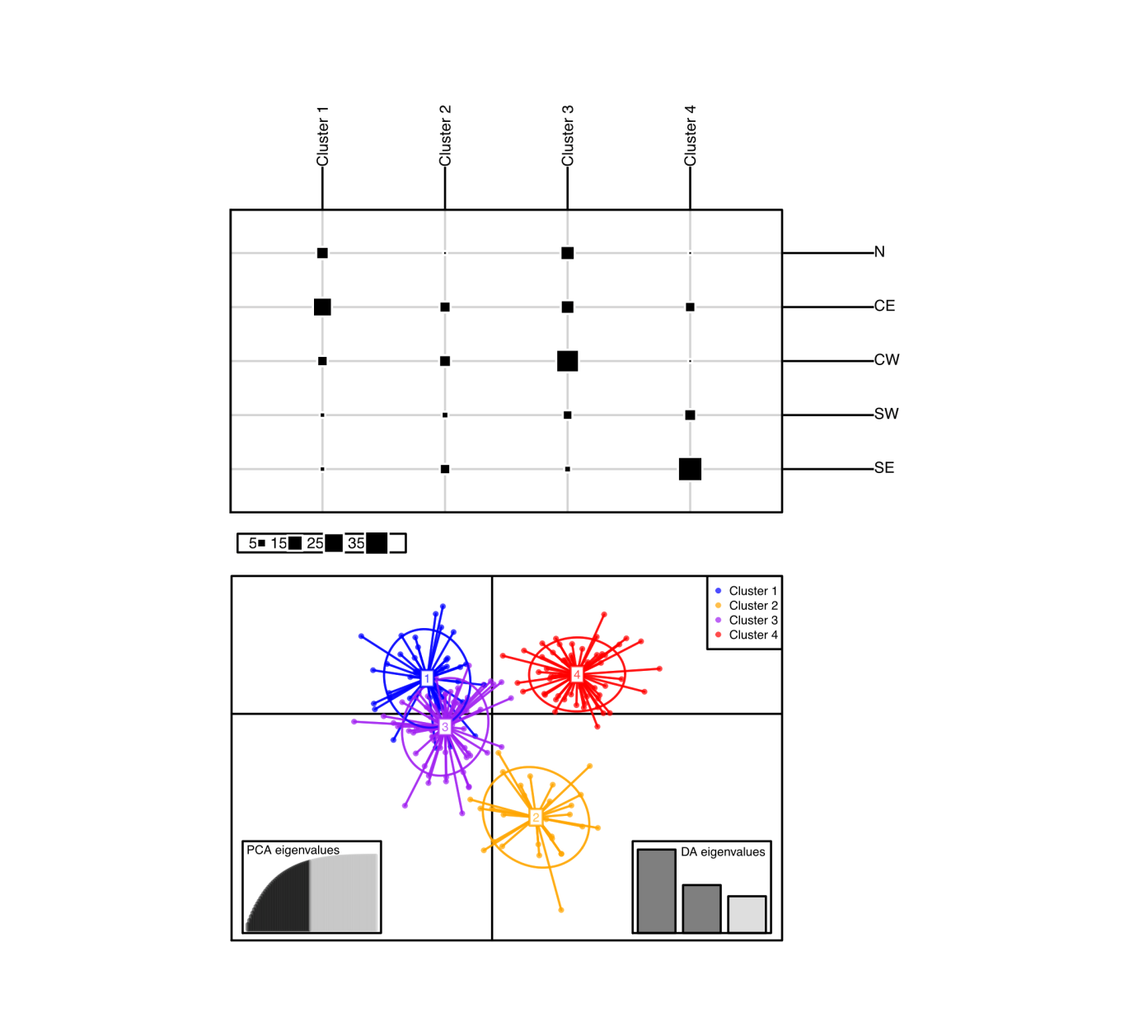


Figure S3. Discriminant analysis of principal components (DAPC) for a reduced dataset of 195 individuals with more equal sample size across groups which shows considerable overlap among two clusters. The axes represent the first two Linear Discriminants (LD). Each dot represents an individual. Numbers represent the different populations identified by DAPC analysis. Geographic groups: N-North; CE-Central-East; CW-Central-West; SW-South-West; SE-South-East.

Table S1. Distribution of 89 haplotypes in Europe (Portugal, Spain, France, Germany, Switzerland, Italy and Serbia). Each haplotype was observed from 1 to 69 times.

| Haplotype | Portugal | Spain | France | Germany | Switzerland | Italy | Serbia | Acc.No. |
| --- | --- | --- | --- | --- | --- | --- | --- | --- |
| H1 | 0 | 16 | 0 | 0 | 0 | 0 | 0 | AY625732.1 |
| H2 | 1 | 3 | 0 | 0 | 0 | 0 | 0 | AY625733.1 |
| H3 | 4 | 5 | 0 | 0 | 0 | 0 | 0 | AY625734.1 |
| H4 | 0 | 2 | 0 | 0 | 0 | 0 | 0 | AY625735.1 |
| H5 | 0 | 2 | 0 | 0 | 0 | 0 | 0 | AY625736.1 |
| H6 | 4 | 9 | 0 | 0 | 0 | 0 | 0 | AY625737.1 |
| H7 | 0 | 1 | 0 | 1 | 0 | 0 | 0 | AY625738.1 |
| H12 | 0 | 0 | 0 | 0 | 0 | 9 | 0 | AY625743.1 |
| H13 | 0 | 0 | 0 | 0 | 0 | 58 | 0 | AY625744.1 |
| H14 | 0 | 0 | 0 | 0 | 1 | 63 | 0 | AY625745.1 |
| H15 | 0 | 0 | 0 | 0 | 0 | 69 | 0 | AY625746.1 |
| H16 | 0 | 0 | 0 | 2 | 9 | 17 | 0 | AY625747.1 |
| H17 | 0 | 0 | 0 | 0 | 0 | 10 | 0 | AY625748.1 |
| H18 | 0 | 0 | 0 | 0 | 2 | 3 | 0 | AY625749.1 |
| H19 | 0 | 0 | 4 | 0 | 0 | 0 | 0 | AY625750.1 |
| H21 | 0 | 0 | 2 | 1 | 0 | 0 | 0 | AY625752.1 |
| H22 | 0 | 0 | 2 | 0 | 0 | 0 | 0 | AY625753.1 |
| H23 | 0 | 0 | 0 | 0 | 8 | 3 | 2 | AY625754.1 |
| H24 | 0 | 0 | 0 | 2 | 10 | 8 | 0 | AY625755.1 |
| H25 | 0 | 0 | 0 | 0 | 11 | 13 | 0 | AY625756.1 |
| H27 | 0 | 0 | 0 | 0 | 0 | 25 | 8 | AY625758.1 |
| H28 | 0 | 0 | 0 | 0 | 0 | 2 | 7 | AY625759.1 |
| H29 | 0 | 0 | 0 | 0 | 0 | 5 | 1 | AY625760.1 |
| H30 | 0 | 0 | 0 | 0 | 0 | 6 | 1 | AY625761.1 |
| H31 | 0 | 0 | 0 | 0 | 0 | 2 | 0 | AY625762.1 |
| H35 | 0 | 0 | 0 | 0 | 0 | 15 | 0 | AY625766.1 |
| H37 | 0 | 0 | 0 | 0 | 0 | 1 | 1 | AY625768.1 |
| H40 | 0 | 0 | 0 | 0 | 0 | 9 | 0 | AY625771.1 |
| H41 | 0 | 0 | 0 | 6 | 11 | 13 | 0 | AY625772.1 |
| H42 | 0 | 0 | 0 | 0 | 0 | 2 | 0 | AY625773.1 |
| H43 | 0 | 0 | 0 | 0 | 0 | 4 | 0 | AY625774.1 |
| H45 | 0 | 0 | 0 | 0 | 0 | 2 | 0 | AY625776.1 |
| H47 | 0 | 0 | 0 | 1 | 1 | 0 | 1 | AY625778.1 |
| H48 | 0 | 0 | 0 | 1 | 0 | 2 | 2 | AY625779.1 |
| H54 | 0 | 0 | 0 | 1 | 2 | 1 | 1 | AY625785.1 |
| H57 | 0 | 0 | 0 | 0 | 0 | 2 | 0 | AY625788.1 |
| H60 | 0 | 0 | 0 | 0 | 0 | 6 | 0 | AY625791.1 |
| H67 | 0 | 0 | 0 | 0 | 19 | 2 | 0 | AY625798.1 |
| H69 | 6 | 0 | 0 | 0 | 0 | 0 | 0 | AY625800.1 |
| H70 | 7 | 0 | 0 | 0 | 0 | 0 | 0 | AY625801.1 |
| H71 | 1 | 1 | 0 | 0 | 0 | 0 | 0 | AY625802.1 |
| H74 | 0 | 2 | 0 | 0 | 0 | 0 | 0 | AY625805.1 |
| H80 | 0 | 0 | 0 | 0 | 0 | 0 | 9 | AY625811.1 |
| H82 | 0 | 0 | 0 | 0 | 0 | 0 | 6 | AY625813.1 |
| H83 | 0 | 0 | 0 | 0 | 3 | 0 | 8 | AY625814.1 |
| H84 | 0 | 0 | 0 | 0 | 0 | 0 | 19 | AY625815.1 |
| H85 | 0 | 0 | 0 | 0 | 0 | 0 | 6 | AY625816.1 |
| H86 | 0 | 0 | 0 | 0 | 0 | 0 | 10 | AY625817.1 |
| H87 | 0 | 0 | 0 | 0 | 0 | 0 | 2 | AY625818.1 |
| H89 | 0 | 0 | 0 | 0 | 0 | 0 | 3 | AY625820.1 |
| H90 | 0 | 0 | 0 | 0 | 0 | 0 | 3 | AY625821.1 |
| H92 | 0 | 6 | 0 | 0 | 0 | 0 | 0 | AY625823.1 |
| H95 | 0 | 0 | 2 | 0 | 0 | 0 | 0 | AY625826.1 |
| H97 | 0 | 0 | 2 | 0 | 8 | 0 | 0 | AY625828.1 |
| H102 | 0 | 0 | 0 | 0 | 0 | 0 | 6 | AY625833.1 |
| H103 | 0 | 0 | 0 | 0 | 0 | 0 | 13 | AY625834.1 |
| H104 | 0 | 0 | 0 | 0 | 0 | 0 | 3 | AY625835.1 |
| H107 | 0 | 0 | 0 | 0 | 0 | 0 | 4 | AY625838.1 |
| H109 | 0 | 0 | 0 | 0 | 0 | 0 | 2 | AY625840.1 |
| H111 | 0 | 0 | 0 | 0 | 0 | 0 | 8 | AY625842.1 |
| H115 | 0 | 0 | 0 | 0 | 0 | 0 | 5 | AY625846.1 |
| H116 | 0 | 0 | 0 | 0 | 0 | 0 | 9 | AY625847.1 |
| H117 | 0 | 0 | 0 | 0 | 0 | 0 | 9 | AY625848.1 |
| H118 | 0 | 0 | 0 | 0 | 0 | 0 | 2 | AY625849.1 |
| H119 | 0 | 0 | 0 | 0 | 0 | 0 | 4 | AY625850.1 |
| H120 | 0 | 0 | 0 | 0 | 0 | 0 | 4 | AY625851.1 |
| H121 | 0 | 0 | 0 | 0 | 0 | 0 | 4 | AY625852.1 |
| H122 | 0 | 0 | 0 | 0 | 0 | 0 | 2 | AY625853.1 |
| H131 | 0 | 0 | 0 | 0 | 0 | 0 | 2 | AY625862.1 |
| H132 | 0 | 0 | 0 | 0 | 0 | 0 | 2 | AY625863.1 |
| H137 | 0 | 0 | 0 | 0 | 0 | 0 | 2 | AY625868.1 |
| H138 | 0 | 0 | 0 | 0 | 0 | 0 | 3 | AY625869.1 |
| H141 | 0 | 0 | 0 | 0 | 0 | 0 | 2 | AY625872.1 |
| H145 | 0 | 0 | 0 | 0 | 0 | 0 | 2 | AY625876.1 |
| H158 | 0 | 2 | 0 | 0 | 0 | 0 | 0 | AY625889.1 |
| H159 | 0 | 4 | 0 | 0 | 0 | 0 | 0 | AY625890.1 |
| H162 | 0 | 0 | 0 | 0 | 6 | 0 | 0 | MW916295 |
| H163 | 0 | 0 | 0 | 0 | 2 | 0 | 0 | MW916296 |
| H164 | 0 | 0 | 0 | 0 | 9 | 0 | 0 | MW916297 |
| H165 | 0 | 0 | 0 | 0 | 3 | 0 | 0 | MW916298 |
| H166 | 0 | 0 | 0 | 0 | 9 | 0 | 0 | MW916299 |
| H167 | 0 | 0 | 0 | 0 | 18 | 0 | 0 | MW916300 |
| H168 | 0 | 0 | 0 | 0 | 1 | 0 | 0 | MW916301 |
| H169 | 0 | 0 | 0 | 0 | 1 | 0 | 0 | MW916302 |
| H170 | 0 | 0 | 0 | 0 | 1 | 0 | 0 | MW916303 |
| H171 | 0 | 0 | 0 | 0 | 1 | 0 | 0 | MW916304 |
| H172 | 0 | 0 | 0 | 0 | 1 | 0 | 0 | MW916305 |
| H173 | 0 | 0 | 0 | 0 | 1 | 0 | 0 | MW916306 |
| H174 | 0 | 0 | 0 | 0 | 0 | 1 | 0 | OL631605 |
